# Health-Associated Niche Inhabitants as Oral Probiotics: The Case of *Streptococcus dentisani*

**DOI:** 10.3389/fmicb.2017.00379

**Published:** 2017-03-10

**Authors:** Arantxa López-López, Anny Camelo-Castillo, María D. Ferrer, Áurea Simon-Soro, Alex Mira

**Affiliations:** Department of Health and Genomics, Center for Advanced Research in Public Health, FISABIO FoundationValencia, Spain

**Keywords:** probiotics, dental caries, pH buffering, arginolytic pathway, bacteriocins, *Streptococcus dentisani*

## Abstract

Oral diseases, including dental caries and periodontitis, are among the most prevalent diseases worldwide and develop as a consequence of a microbial dysbiosis. Several bacterial strains are being tested as potential oral health-promoting organisms, but usually they are species isolated from niches other than the site where they must exert its probiotic action, typically from fecal samples. We hypothesize that oral inhabitants associated to health conditions will be more effective than traditional, gut-associated probiotic species in key aspects such as colonization of the oral site where disease takes place or the possession of oral health promoting functions, as well as more practical issues like safety and toxicity, and establishing proper doses for administration. As an example of these *active colonizers*, we describe the case of *Streptococcus dentisani*, a new streptococcal species isolated from dental plaque of caries-free individuals. We have detected it in 98% of dental plaque samples from healthy individuals and, as expected, it does not produce any toxic secondary metabolite and does not survive a simulated stomach digestion, preventing potential secondary effects. Besides, this species has a double probiotic action, as it inhibits the growth of major oral pathogens through the production of bacteriocins, and also buffers acidic pH (the primary cause of dental caries) through an arginolytic pathway. We propose the use of *S. dentisani* as a promising probiotic against tooth decay.

## Introduction

Oral diseases such as dental caries (tooth decay) and periodontitis (gum disease) are caused by microorganisms. However, they are currently not considered infectious diseases in classical terms because their etiology is clearly polymicrobial (Fejerskov, 2004; Simón-Soro and Mira, 2015), and because the pathogenic bacteria involved are also found at lower proportions in healthy individuals (Hajishengallis and Lamont, 2012; Camelo-Castillo et al., 2015). Thus, it has been pointed out that antimicrobial strategies may not be effective against oral diseases, and new preventive or therapeutic approaches based on restoring the microbial ecological balance in the oral cavity have been proposed (Marsh, 2015; Marsh et al., 2015). Those new preventive measures could include the use of prebiotic compounds to promote the growth of health-associated bacteria (Santarpia et al., 2014), or the application of probiotic bacteria with beneficial features (Saha et al., 2012). In the case of dental caries, health-associated microbial communities have been identified using omics approaches (Zaura et al., 2009; Belda-Ferre et al., 2011; Alcaraz et al., 2012), and therefore culturing those microorganisms could provide potential beneficial bacteria to prevent oral diseases.

However, due to the greater development of probiotics in gut pathologies, and the strong safety evidence accumulated for gut bacteria, many microorganisms isolated from human or animal fecal samples with beneficial properties are being developed as potential probiotics to promote oral health. These often include strains of lactobacilli and bifidobacteria, which had previously been shown to have inmunomodulatory, anti-inflammatory and anti-bacterial properties in different *in vitro* studies and also in clinical trials (see **Table 1**; Reid et al., 2011; Cagetti et al., 2013). However, the use of many of these strains in oral diseases like dental caries can be problematic because both *Lactobacillus* and *Bifidobacterium* species have been shown to be acidogenic and to be involved in tooth decay (Badet and Thebaud, 2008; Mantzourani et al., 2009a). Even if weak acidogenic strains are selected, the capacity of gut bacteria to colonize the oral niche and to produce anti-caries effects has still to be demonstrated, and the use of gut probiotics in the oral cavity has been criticized. For instance, when the strain *Lactobacillus salivarius* W24 was tested in an *in vitro* oral biofilm model, it was shown that this strain further lowered the pH and affected the compositional stability of oral communities (Pham et al., 2009). Thus, the identification of novel strains isolated from the oral cavity itself could be instrumental for the development of efficient probiotics applied to oral health.

**Table 1:** Bacteria tested in clinical trials as oral care probiotics.

Strain	Origin	Brand	Disease	Reference^1^
*Lactobacillus rhamnosus* GG	Human intestinal tract		Caries	Aminabadi et al., 2011
*Lactobacillus rhamnosus* LB21	Human intestinal tract		Caries	Stecksén-Blicks et al., 2009
*Lactobacillus reuteri* 55730 + PTA 5289	Woman breast milk	Prodentis^TM^	Ginvival health, plaque reduction and caries	Krasse et al., 2006; Caglar et al., 2008a
*Lactobacillus brevis* CD2	Dairy products	Inersan^®^	Periodontal disease and caries	Riccia et al., 2007; Campus et al., 2014
*Lactobacillus paracasei* SD1	Human oral cavity		Caries	Ritthagol et al., 2014
*Bifidobacterium animalis* DN-173 010	Dairy products	ACTIVIA^®^	Caries	Caglar et al., 2005
*Bifidobacterium animalis subsp. lactis* BB-12^®^	Dairy cultures	BB-12^®^	Caries	Caglar et al., 2008b
*Streptococcus salivarius* M18	Saliva of a healthy child	BLIS M18^®^	Plaque reduction	Burton et al., 2013
*Lactobacillus salivarius* WB21	Mammalian digestive tract		Gingival health, periodontal disease, halitosis and caries	Shimauchi et al., 2008; Mayanagi et al., 2009; Iwamoto et al., 2010; Suzuki et al., 2014
*Streptococcus oralis* KJ3^®^, *Streptococcus uberis* KJ2^®^ and *Streptococcus rattus* JH145^®^	Healthy mouths	ProBiora(3)^®^	Caries, periodontal disease and halitosis	Zahradnik et al., 2009
*Weissella cibaria* CMU, CMS-1, CMS-2 and CMS-3	Children’s saliva		Halitosis and plaque reduction	Kang et al., 2006a,b
*Lactobacillus reuteri* DSM 17938 + PTA 5289	Human saliva	BioGaia ProDentis	Halitosis	Keller et al., 2012
*Lactobacillus plantarum* 7481 and *Lactobacillus brevis* 7480	Children’s saliva	AB:Dentis^®^	Halitosis and gingivitis	WO 2012022773 A1^∗^
*Streptococcus salivarius* K12	Children’s saliva	BLIS K12^®^	Halitosis	Burton et al., 2006
*Lactobacillus acidophilus* NK1 + 100ash + K3III24	Human intestinal tract	Acilact (solely Russia)	Periodontal disease	Pozharitskaia et al., 1994
*Bifidobacterium bifidum* N1 and *Lactobacillus acidophilus* NK1	Human intestinal tract	Bifidumbacterin + Acilact (solely Russia)	Periodontal disease	Grudianov et al., 2002
*Lactobacillus casei* 37	Human intestinal tract		Periodontal disease	Volozhin et al., 2004
*Lactobacillus paracasei* GMNL-33 (ADP-1)	Human oral cavity	Dental-Lac^TM^	Periodontal disease	Chuang et al., 2011
*Lactobacillus* salivarius TI 2711 (LS 1)	Human saliva		Periodontal disease	Matsuoka et al., 2006
*Lactobacillus rhamnosus* GG (53103) + LC705	Human intestinal tract		Oral candidasis	Hatakka et al., 2007

*^1^Reference = Publication with results of clinical trial; ^∗^Patent number.*

In the current manuscript, we describe the potential probiotic features of the new species *Streptococcus dentisani* that we recently isolated from the dental plaque of caries-free individuals (Camelo-Castillo et al., 2014). There are currently six isolates for which the genome sequence is available and that robust phylogenomic analysis include within the *dentisani* cluster (Jensen et al., 2016). This cluster forms part of the *S. oralis* clade and differs from the *S. oralis* and *S. tigurinus* clusters mainly in their ability to hydrolyze arginine (Jensen et al., *2*016). In this manuscript, we study strains 7746 and 7747^T^, which were isolated from two different individuals and that were shown to be different by comparison of their genomes (Camelo-Castillo et al., 2014). We describe their ability to inhibit the growth of caries-producing bacteria, as *Streptococcus mutans* and *Streptococcus sobrinus*, and to buffer extracellular acidic pH, which is the underlying cause of tooth decay. In addition, we show that, being a commensal species in the oral cavity of healthy individuals, their safety features are robust and the appropriate dose for probiotic treatment can be easily determined experimentally.

## Materials and Methods

### Inhibition Assays with *S. dentisani* Supernatants

Inhibition experiments against cariogenic bacteria were carried out with concentrated supernatants of well grown cultures of *S. dentisani* 7746 and 7747 (stationary phase). In order to obtain 5 ml of the concentrated supernatants, single colonies of each *S. dentisani* strain were inoculated into 50 ml of brain hearth infusion broth (BHI, Biolife) and incubated aerobically at 37°C without agitation during 48 h, or until they reached an optical density of around 1.5 at 610 nm (O.D. _610_). After the incubation period, the cultures were centrifuged at 4000 rpm 10 min and the pellets discarded. The obtained supernatants were filtered throughout 0.2 μm pore-size filters (Millipore) and ten-fold concentrated by rotary evaporation on a RV 10 digital device (VWR) with the following settings: heating bath at 40°C, 70 rpm of rotation, 100 mbar of pressure, and 30 min of operating time. The resulting 5 ml of concentrated supernatants were filtered throughout 0.2 μm pore-size filters (Millipore) and stored at –20°C until use.

The inhibitory activity of the supernatants was determined by monitoring the growth of *S. mutans* ATCC 25175 and *S. sobrinus* CECT 4034 in the presence/absence of the potential inhibitor by means of optical density measurements. Pre-inocula of *S. mutans* and *S. sobrinus* were obtained by inoculating a single colony of each strain in 10 ml of BHI liquid medium and incubated aerobically overnight at 37°C without agitation. The O.D. _610_ of the pre-inocula was measured in a spectrophotometer (BioPhotometer, Eppendorf) and diluted with BHI liquid medium to obtain an optical density of 0.1. To assess the inhibitory effect of *S. dentisani* on the growth of *S. mutans* and *S. sobrinus*, 160 μl of the bacterial suspensions were mixed with 40 μl of the concentrated supernatants and loaded by triplicate into a Nunc Microwell 96-well microplate by triplicate. As controls, 160 μl of the bacterial suspensions were mixed with 40 μl of 10-fold concentrated BHI and loaded by triplicate into the 96-well microplate. The microplate was loaded into a microplate reader (Infinite 200 PRO, Tecan) and incubated at 37°C during 24 h. The O.D. _610_ of each inoculated well was measured every 30 min during the incubation time.

To determine the active size fraction, the concentrated supernatant was size-fractionated by sequential filtering throughout 10 KDa and 3 KDa filters (Amicon), following the manufacturer’s recommendations. By this way, we obtained three size fractions (>10 KDa, 3–10 KDa, and <3KDa) that were tested by triplicate against *S. mutans* cultures by the same methodology explained above. To confirm the peptidic nature of the inhibitory compounds and discard that the inhibition was produced by hydrogen peroxide we proceed as described in Zhu and Kreth (2012). Briefly, *S. dentisani* was grown on a BHI plate for 24 h and peroxidase (40 μg), peptidase (64 μg), or phosphate-buffered saline were applied beside each colony for 10 min before the other species were inoculated at the same spot. *S. mutans* and *S. sanguinis* were used as controls of proteinaceus inhibitory substance and H_2_O_2_, respectively.

Besides, we used scanning electron microscopy (SEM) to directly observe the effect of the *S. dentisani* supernatants on the three type strains *S. mutans* ATCC 25175, *Prevotella intermedia* NCTC 13070, and *Fusobacterium nucleatum* DSMZ 20482 cells. Briefly, 160 μl of the bacterial cultures in exponential phase (O.D. _610_ = 0.6) were mixed with 40 μl of the concentrated supernatant and incubated for 30, 60, and 90 min at 37°C. After the incubation period, the suspensions were centrifuged at 4000 rpm 10 min and the supernatant discarded. The pellets were fixed in 2.5% paraformaldehyde and 0.5% glutaraldehyde, washed three times with PBS buffer and exposed to 1% osmium tetroxide in PBS buffer for 1 h. The samples were rinsed with PBS buffer three more times and then moved through a gradual process of dehydration, starting with 30% ethanol and ending with absolute ethanol (multiple rinse steps at each 30, 50, 70, 80, 90, and 100% ethanol). Finally, the samples were mounted on scanning electron micrograph stubs, sputter coated with gold, and viewed on a JEOL JSM 840 scanning electron microscope.

### pH Range of Growth and Resistance of *S. dentisani* to the Digestive Process

To know the optimal and pH growth range of *S. dentisani* strains 7746 and 7747 we prepared BHI broth at different pHs (4.7, 5.5, 6, 6.5, 7, and 7.5) by adding NaOH 10 N or HCl 10 N to the commercial medium (Biolife). Pre-inocula were obtained as explained above. Two-hundred microliters of the BHI broths at different pHs were inoculated by triplicate with 20 μl of the inocula into a Nunc Microwell 96-well microplate. The O.D. _610_ of each inoculated well was measured every 2 h and plotted versus the time of incubation to obtain the growth curves.

To evaluate the resistance of *S. dentisani* to the digestive process, the viability of the strains 7746 and 7747 was checked after a treatment with the salivary enzyme alpha amylase, followed by digestion with gastric enzymes under progressive acidic conditions to simulate digestion, following the protocol described in Khalf et al. (2010) and Martínez et al. (2011). The assays were performed in an i*n vitro* model stomach (or dynamic digester) by the external services of the Food Industry Research Association AINIA (Valencia, Spain). Each strain was tested by duplicate whereas the control was assayed once. Colony forming units (CFU) counts were obtained in BHI agar before any treatment (reference cultures), after the chewing step, and during the gastric digestion at three different times (30, 60, and 120 min). The plates were incubated at 37°C during 24–48 h. As the medium used for CFU counts was not selective for *S. dentisani*, the same assay was made without inoculation and used as blank to discount any contamination, obtaining 0 CFUs in this control.

### Growth of *S. dentisani* in an Arginine Enriched Medium

*Streptococcus dentisani* 7746 was grown in 300 ml of BHI medium amended with 5 g/l of L-arginine monohydrochloride 98% (Alfa-Aesar). The pre-inoculum was obtained by inoculating a single colony of the strain in 10 ml of BHI broth and incubating overnight at 37°C without agitation. Inoculation was made with the volume required to obtain an initial O.D. _610_ of 0.02. For comparison, the same volume was used to inoculate 300 ml of BHI without arginine. In both conditions the initial pH was set at 7.3. The cultures were incubated at 37°C during 24 h and aliquots of 1 ml were taken every hour for measuring the pH and the O.D. _610_. Both conditions were assayed by triplicate.

### Prevalence of *S. dentisani* and Other Oral Probiotics in the Dental Plaque of Healthy Individuals

To assess the prevalence of *S. dentisani* in the dental plaque of healthy individuals we compared the genomic sequences of the *S. dentisani* strains 7746 and 7747 (1.98 and 1.74 Mb, respectively) against 118 metagenomes of the dental plaque of healthy individuals available at The Human Microbiome Project Consortium (HMP, Turnbaugh et al., 2007). The metagenomic reads were mapped against the sequenced reference genomes using the NUCmer and PROmer v3.06 alignment algorithms (Kurtz et al., 2004) with the default parameters, following the methodology of Belda-Ferre et al. (2011). We considered that *S. dentisani* was present in a sample if at least 20 sequences >100 bp of the metagenome showed a similarity equal to or higher than 99% with the sequenced genomes. For comparison, the same approach was used to analyze the prevalence of *S. salivarius* in dental plaque.

The presence of the genus *Lactobacillus* in different parts of the oral cavity was performed by the analysis of the oral 16S rDNA sequences deposited in the Human Microbiome Project database in year 2014^1^. The niches analyzed were the keratinized gingiva, buccal mucosa, hard palate, palatine tonsils, saliva, supra- and subgingival plaque, and tongue dorsum. The analyses were carried out with a total of 4.3 × 10^8^ sequences. The taxonomic affiliation was performed using the Megablast tool implemented in the NCBI against the SILVA ribosomal database^2^, with the following parameters: *e* value <1e–^10^, percent identity >97%, alignment length >350 bp.

### Quantification of *S. dentisani* in the Dental Plaque of Healthy Individuals

Two healthy volunteers, named MG01 and MG02, were selected for sampling. They were males aged 20–30 years, non-smokers, with 28 teeth excluding third molars, with good dental and periodontal health: in both, absence of caries (non-cavitated level), DMF = 0, OHI = 0, GI = 1, and CPI = 1 (following nomenclature by World Health Organization [WHO], 1997). They had not been treated with antibiotics in the 6 months prior to the study nor presented antecedents of routine use of oral antiseptics. The two donors signed a written informed consent and the sampling procedure was approved by the Ethics Committee from the DGSP-CSISP (Valencian Health Authority), with reference 10/11/2009. Supragingival and subgingival dental plaque samples were taken from vestibular (buccal) and lingual (palatine) surfaces of 28 teeth in each volunteer. *Streptococcus dentisani* was quantified in individual free surfaces of each tooth type (incisor, canine, premolar, and molar) from one quadrant and the absolute numbers calculated by multiplying the obtained value by the number of each tooth type in the mouth. The same procedure was followed for the total bacterial content quantification.

Dental plaque samples were resuspended in 100 μl of PBS buffer and DNA was extracted with the MagnaPure LC JE379 instrument and the MagnaPure LC DNA Isolation Kit (Roche). The quantification of the DNA was done with the Quant-iT PicoGreen dsDNA Assay Kit (Invitrogen), and real-time PCR was performed in a LightCycler 480 System with the LightCycler 480 SYBR Green I Master Mix (Roche). In every step, we followed the manufacturer’s recommendations.

The specific primers used for the quantification of *S. dentisani* were designed for this study and targeted the genes for the carbamate kinase (*arcC*): CkSdF 5′-GTAACCAACCGCCCAGAAGG-3′ and CkSdR 5′-CCGCTTTCGGACTCGATCA-3′; and the ORF540: Orf540F (5′-ATGTTCATCGGCTTGACAGGCTT-3′) and Orf540R (5′- TAAGCAAGCATAGAACCGCGCC-3′). Primers specificity was predicted *in silico* by the primerBLAST tool implemented in the NCBI^3^ and confirmed by absence of amplification by qPCR with 5 ng/μl DNA from *S. mutans, S. sobrinus, S. sanguinis, S. salivarius, S. mitis, S. pneumoniae, S. infantis, and S. oralis*. The specificity of the primers was also checked by scrutinizing the melting profiles after every assay. Primers for the Ck gene did not amplify any of the tested streptococcal species. Primers for the ORF540 amplified only the DNA of *S. pneumoniae*, which is a rare inhabitant of dental plaque. Amplification was performed in a 20 μl final volume containing 1 μl of template DNA (at concentrations 5–22 ng/μl), 10 μl of the LightCycler 480 SYBR Green I Master Mix, 0.4 μl of each primer, and 7.2 μl of nuclease-free water. The thermocycling protocol used was as follows: an initial step of 95°C for 5 min, and 40 cycles of 10 s at 95°C, 20 s at 65°C, and 25 s at 72°C. All the quantifications were made by duplicate.

The concentration of *S. dentisani* in each sample was calculated by comparison with the Cq values obtained from a standard curve. This was generated using serial 10-fold dilutions of DNA extracted from 2 × 10^7^ CFUs/ml (counted by serial dilutions in agar plates). Finally, the Cq values obtained with the plaque samples were replaced in the standard equation and expressed in absolute numbers per tooth type analyzed.

## Results

### Inhibitory Activity of the *S. dentisani* Supernatants

**FIGURE 1:**
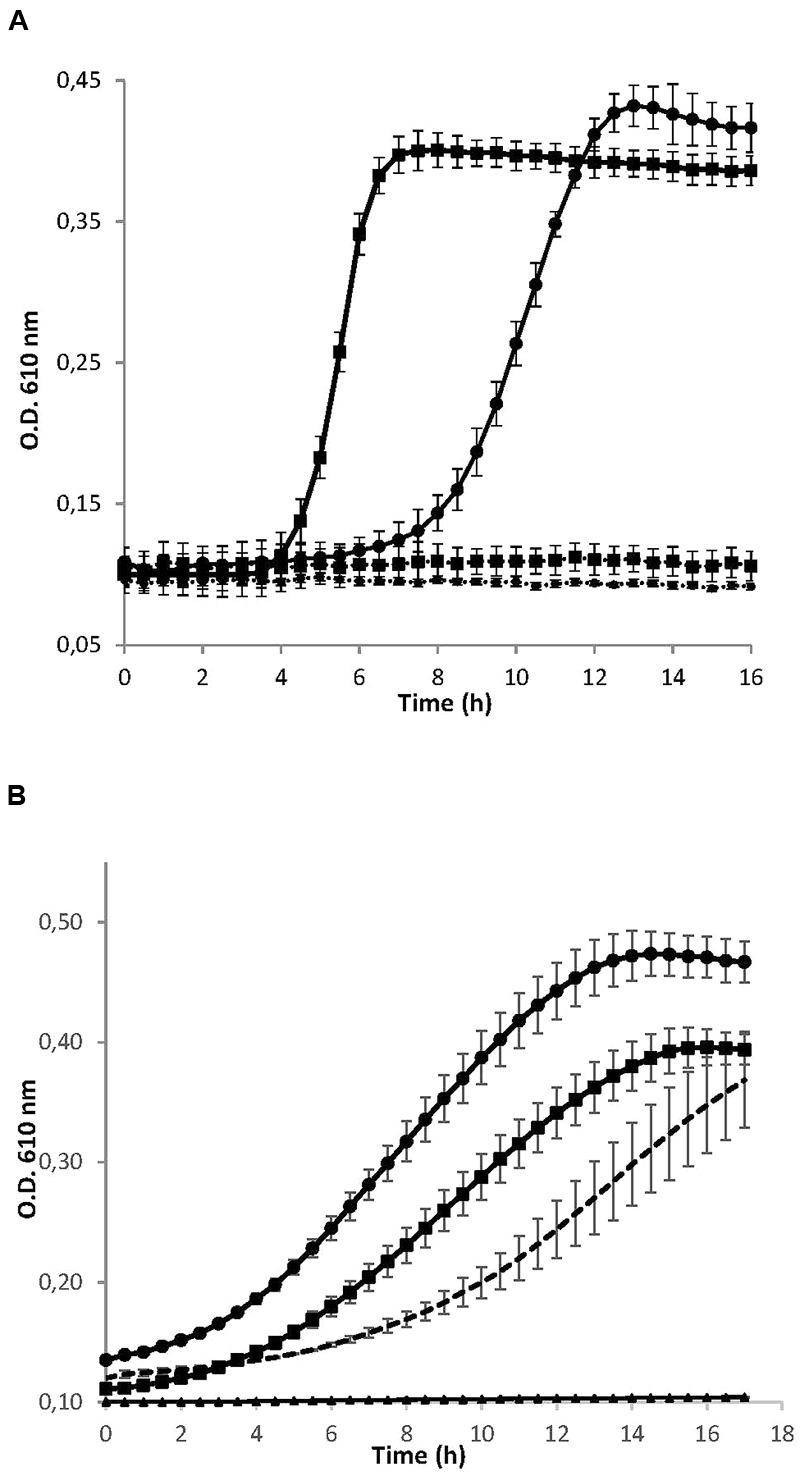
**(A)** Growth curves of the cariogenic bacteria *Streptococcus mutans* ATCC 25175 (squares) and *S. sobrinus* CECT 4034 (circles) in the presence (dotted lines) and absence (solid lines) of concentrated supernatant of *S. dentisani* strain 7746. **(B)** Growth curves of *S. mutans* ATCC 25175 in the presence of different size fractions of the 10× concentrated supernatant of *S. dentisani* 7746. Circles correspond to the fraction >10 KDa, squares to the 3–10 KDa fraction, and triangles to the fraction <3 KDa. For comparisons, *S. mutans* was grown in the presence of 10x concentrated BHI medium (dotted line). Means ± SD from three independent replicates are plotted.

As shown in **Figure 1A**, the addition of the *S. dentisani* 7746 concentrated supernatant produced a marked inhibitory effect on the growth of both *S. mutans* and *S. sobrinus*, as compared to the curves obtained when only concentrated BHI was added. Similarly, the concentrated supernatant completely inhibited the growth of other *S. mutans* strains (strains OMZ175 and ATCC 700610, data not shown). Regarding the curves obtained with the different size fractions, the <3 KDa fraction retained the inhibitory activity when tested against *S. mutans* ATCC 25175, while the 3–10 KDa and >10 KDa fractions did not (**Figure 1B**). The last two even enhanced the growth of *S. mutans*, probably due to a higher availability of nutrients coming from the concentrated culture medium and/or the *S. dentisani* metabolism. The same results were obtained with strain 7747 (data not shown).

**FIGURE 2:**
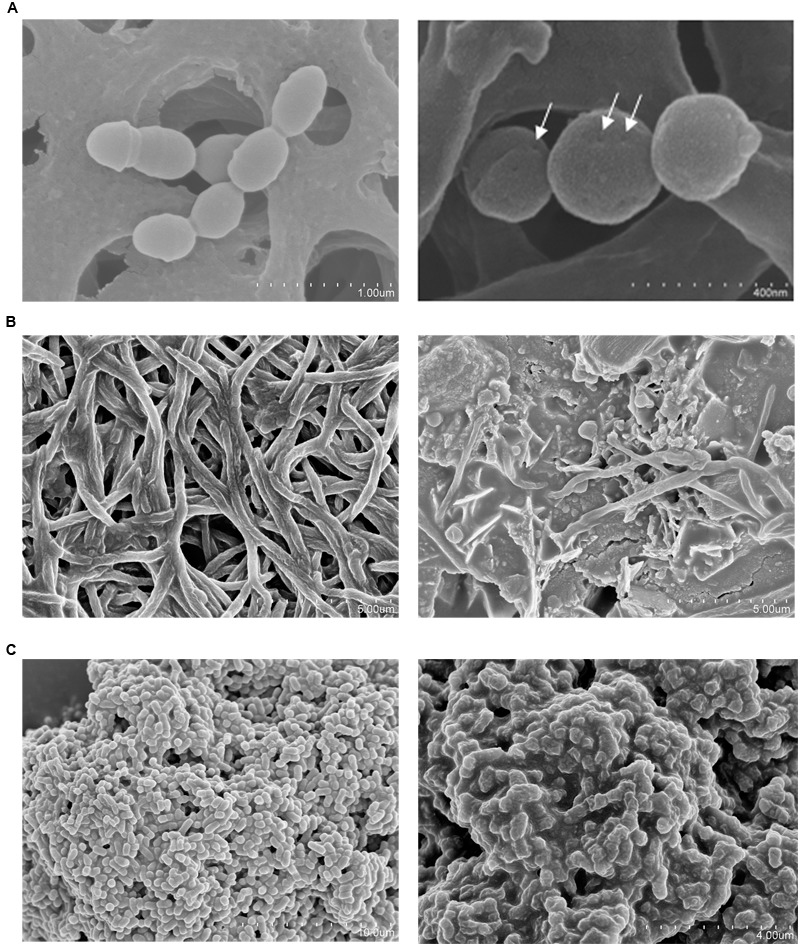
**Scanning electron microscopy (SEM) of three oral pathogens before (left) and after (right) a 30-min treatment with 10-fold concentrated supernatant of *S. dentisani* strain 7746. (A)** *Streptococcus mutans* (pores are pointed with arrows). **(B)** *Fusobacterium nucleatum*. **(C)** *Prevotella intermedia*.

Microscopy visualizations revealed that after 30 min of incubation with concentrated supernatants of *S. dentisani*, the cells of *S. mutans, F. nucleatum*, and *P. intermedia* were clearly affected, showing pores in the surface of the cells (*S. mutans*), changes in the cell walls’ structure (*P. intermedia*) and cell lysis (*F. nucleatum*) (**Figures 2A–C**).

**FIGURE 3:**
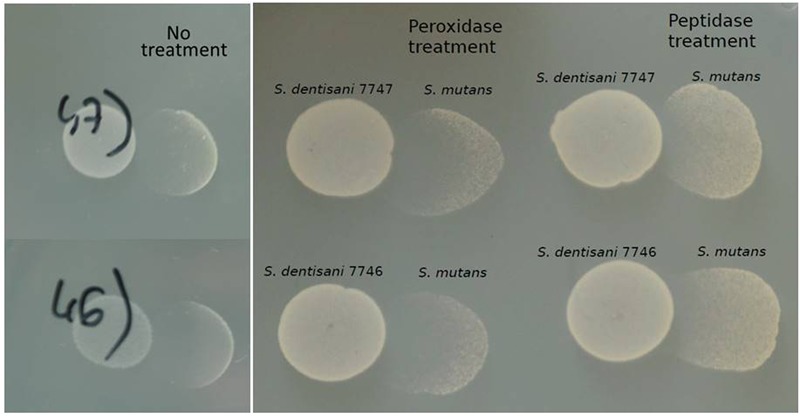
**Inhibition of *S. mutans* by *S. dentisani* in the presence of peroxidase and proteinase.** The tests were performed with *S. dentisani* strains 7746 and 7747, treating with the enzymes the spots where *S. mutans* would later be grown. The controls on the left panel show the inhibition without treatment.

Proteinase treatment of the supernatant provoked the absence of growth inhibition when tested against *S. mutans*, while the peroxidase treatment did not affect the inhibitory activity (**Figure 3**). Taken together, the results indicated that the inhibition is not due to the production of hydrogen peroxide, and supports the idea that *S. dentisani* inhibit oral pathogenic bacteria by the production of inhibitors of peptidic nature, such as bacteriocin-like peptides.

### pH Tolerance and Resistance of *S. dentisani* to the Digestive Process

*Streptococcus dentisani* was tested for its ability to grow at different pHs. The growth curves obtained showed that both strains displayed a very similar behavior, with an optimal growth pH close to 7 (**Supplementary Figures S1A,B**). It is noteworthy that they were able to grow at pH values between 6 and 7.5 but not between 4.7 and 5.5, indicating that *S. dentisani* can endure moderately acidic conditions but is not an acidophilic organism. Surprisingly, the growth of both strains at pH 6 was better than at 6.5, suggesting the activation of a buffering metabolic pathway at pH values around 6 (see Section “pH Buffering Capacity of *S. dentisani*” below).

Regarding the simulated digestive process, and as expected from the pH curves obtained before, *S. dentisani* was not able to maintain the initial viability during digestion simulations. As starting values of viability we obtained 6.5 × 10^8^ and 3.9 × 10^8^ CFUs/ml for the strains 7746 and 7747, respectively. After the chewing simulation with salivary enzymes these values decreased in only two to three orders of magnitude, showing that both strains could remain in the mouth at high levels after chewing, whereas the gastric digestion strongly inhibited their growth, even after the first step of the simulation (30 min, see **Table 2**). As neither strain of *S. dentisani* survived the gastric process, the simulation of an intestinal digestion was not performed.

**Table 2:** Viability, expressed in CFU/ml, of the two strains of *S. dentisani* after the chewing and gastric digestion processes (G.D.) at three different times.

Strain	Starting culture	Chewing pH 6.9	G.D. 30 min pH 3	G.D. 60 min pH 2.1	G.D. 120 min pH 1.7
*S. dentisani* 7746	6.5 × 10^8^± 3.05 × 10^8^	5.3 × 10^6^± 2.8 × 10^8^	<10	<10	<10
*S. dentisani* 7747	3.9 × 10^8^± 4 × 10^7^	1 × 10^6^± 9.8 × 10^5^	<10	<10	<10

*The values shown are the mean of two independent replicates and the standard deviation is specified.*

### pH Buffering Capacity of *S. dentisani*

**FIGURE 4:**
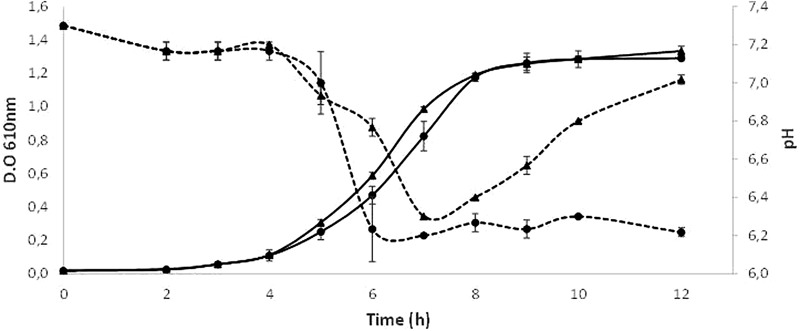
**Growth curves of *S. dentisani* strain 7746 in a medium with (triangles) and without (circles) the addition of 5 g/l of arginine.** The mean ± SD of O.D. _610_ (solid lines) and pH of the culture (dotted lines) from three replicates are depicted.

**Figure 4** depict the growth curves obtained by triplicate when *S. dentisani* 7746 was grown in presence/absence of arginine. During the initial 6 h of incubation, the growth seemed to be fueled by the sugars present in the BHI medium, as the growth curves were identical in both conditions (with and without arginine), and the culture pH dropped from 7.3 to about 6.2. Soon after completion of this first phase, in the cultures containing arginine the pH starts to rise, reaching almost the initial pH value 12 h after inoculation. Contrarily, in the medium without arginine the pH continuously decreased to reach a value of about 6.2. In both conditions the O.D. _610_ measurements were similar, with a maximum value of around 1.3 after 12 h of incubation.

The analysis of the genome of *S. dentisani* showed that strains 7746 and 7747^T^ contain the key genes of the arginine deiminase system: *arcA* (arginine deiminase, locus tag HK29_RS02275), *arcC* (carbamate kinase, locus tag HK29_RS02285), and *arcB* (ornithine carbamoyltransferase, locus tag HK29_RS02280), involved in the ammonia generation through arginine metabolism, a mechanism that releases ammonnia extracellularly producing the alkalinization of the environment (Liu et al., 2008, 2012).

### Prevalence of *S. dentisani* in the Dental Plaque of Healthy Individuals

The recruitment analyses showed that sequences of *S. dentisani* were present in most of the analyzed metagenomes (direct, whole-sequenced DNA coming from dental plaques of healthy individuals at the Human Microbiome Project). At least 20 metagenomic sequences >100 bp were found to have a similarity ≥99% compared with the 7746 genome in 116 out of 118 individuals, and in the strain 7747 this threshold was fulfilled in all 118 individuals. Contrarily, the species *S. salivarius* that has been proposed as a probiotic agent against caries was only detected in three of these metagenomes, in agreement with this species inhabiting mucosal surfaces and not the hard tissues. In addition, we have analyzed the *Lactobacillus* 16S rRNA gene sequences from oral samples contained in the HMP database, as this genus contains species commonly used as oral probiotics (namely *L. acidophilus, L. reuteri*, and *L. rhamnosus*). The results showed that *Lactobacillus* spp. is present in very low numbers in the oral cavity, accounting for less than 0.05% of the bacterial genera in the buccal mucosa, hard palate, palatine tonsils, saliva, tongue, and supragingival plaque. The highest numbers of *Lactobacillus* 16S rDNA sequences were obtained in the subgingival plaque, but even here this genus accounted for only 0.94% of the total bacterial population. We analyzed in the same oral niches the prevalence of the genus *Streptococcus*, which was found to be present at very high numbers in the keratinized gingiva, buccal mucosa, hard palatine, palatine tonsil, tongue dorsum, and saliva (59.5, 58.8, 54, 27.1, 26.8, and 19.7 per cent of the total bacterial members, respectively). Furthermore, the percentage of the genus *Streptococcus* in the subgingival and supragingival plaques was high (18.8 and 20.2%, respectively). Overall, the results showed that the genus *Streptococcus* is much more abundant than *Lactobacillus* in every analyzed oral niche, and particularly in those directly affected by the cariogenic processes, being *Streptococcus* between 20 and 600 times more abundant than *Lactobacillus* in the subgingival and supragingival plaque, respectively.

### Quantification of *S. dentisani* in the Dental Plaque of Healthy Individuals by q-PCR

The absolute numbers of *S. dentisani* cells were obtained for the teeth’s free surfaces of two healthy volunteers by the use of two set of *S. dentisani*-specific primers, which provided similar estimates (**Table 3**). The results showed that both individuals contain very similar values of abundance of *S. dentisani* (1.04 × 10^7^ and 3.14 × 10^7^ cells for MG01 and MG02, respectively, as estimated by the ORF540 primers; 4.46 × 10^7^ and 6.94 × 10^7^ cells for MG01 and MG02, as estimated by the carbamate kinase primers). However, although the calculated *S. dentisani* numbers in the mouth of both individuals was highly similar, its distribution was very different. As shown in **Table 3**, MG01 did not show important differences in the amounts of *S. dentisani* between tooth types nor between the lingual and vestibular surfaces. Patient MG02 however, had a more heterogeneous distribution, with higher proportions of *S. dentisani* in premolars and molars, and on the lingual surfaces of every tooth type.

**Table 3:** Total cell counts of *S. dentisani* on supragingival dental plaque in the vestibular (V) and lingual (L) parts of different tooth types in two caries-free individuals (MG01 and MG02).

	Incisor	Canine	Premolar	Molar
**MG01 (V)** **(L)**	2.1 × 10^5^/2.8 × 10^4^ 2.8 × 10^5^/2.7 × 10^4^	4.7 × 10^5^/7.9 × 10^4^ 1.4 × 10^5^/4.1 × 10^5^	2.9 × 10^5^/3.46 × 10^4^ 4.6 × 10^5^/9.8 × 10^6^	3.1 × 10^5^/3.1 × 10^4^ 4.3 × 10^5^/6.9 × 10^4^
**MG02 (V)** **(L)**	5.5 × 10^2^/7.5 × 10^3^ 3.4 × 10^4^/5.1 × 10^4^	2.7 × 10^3^/4.4 × 10^3^ 2.8 × 10^4^/5.9 × 10^3^	1.2 × 10^3^/1.3 × 10^4^ 7.1 × 10^4^/5.0 × 10^5^	2.5 × 10^4^/4.5 × 10^4^ 4.7 × 10^4^/1.7 × 10^7^

*Data show the estimates of bacterial numbers obtained by qPCR with two different sets of *S. dentisani*-specific primers (orf540/CK).*

## Discussion

Dental caries is considered the most prevalent disease worldwide, with up to 80% of the human population being affected at some point during their lives (Petersen and Lennon, 2004). There are however no efficient means to prevent it, as the polymicrobial nature of the infection and its complex etiology make passive and active immunization strategies (e.g., a caries vaccine) ineffective (Fejerskov, 2004; Simón-Soro and Mira, 2015). Thus, new strategies directed towards the re-establishment of the natural balance in the oral microbiome like the use of pre- and probiotics have been proposed (Marsh et al., 2015). However, as shown in **Table 1**, most of the probiotics proposed to promote oral health are bacteria isolated from environments other than the oral cavity, usually the human gut (Cagetti et al., 2013). In addition, the most commonly used probiotic bacteria to treat dental caries are lactobacilli, due to their well-proven safety characteristics. However, the use of non-oral bacteria may prevent efficient colonization of the oral niche, which is a vital and desirable feature of probiotics. This is supported by the low frequencies of lactobacilli detected in tooth tissues by molecular methods, as reported in the current manuscript. In addition, lactobacilli are important acid-producers and long known to be associated to dental caries lesions (Badet and Thebaud, 2008; Plonka et al., 2012), as well as bifidobacteria (Mantzourani et al., 2009b; Beighton et al., 2010), which are also common oral probiotics. This can be the reason why *in vitro* experiments with these bacteria, even if low-acid producing strains are selected, may result in pH acidification (Pham et al., 2009). These results underline the need to use potential probiotics from the oral cavity, and therefore the isolation of a bacteriocin-producing probiotic *Streptococcus salivarius* which inhibited the cariogenic agent *S. mutans* was promising (Wu et al., 2015). However, the comparison of the *S. salivarius* genome against a high number of metagenomes from supragingival plaque presented in the current manuscript reveals its absence in the tooth, in agreement with its soft-tissues-associated nature. *Streptococcus salivarius* is a typical inhabitant of the buccal epithelium, tongue, and dorsal epithelium (Bowden et al., 1979; Power et al., 2008) and comprises an important part of the total cultivable flora on the soft tissues of the mouth (Wilson, 2005). Probably for this reason, it has been proposed as a probiotic for the pharyngeal mucosa (Guglielmetti et al., 2010) but its inability to colonize the tooth surface may hamper its potential as an anti-caries probiotic. The high heterogeneity of environments in the mouth and the resulting adaptation of microorganisms to those specific microniches (Simon-Soro et al., 2013) underscores the importance of selecting oral bacteria adapted to live in hard tissues as probiotics against dental caries to guarantee a proper colonization. In agreement with this view, our data show that *Streptococcus dentisani*, which was isolated from supragingival dental plaque of caries-free individuals (Belda-Ferre et al., 2011; Camelo-Castillo et al., 2014), was widespread among dental plaque samples from healthy subjects.

Apart from dental colonization, we show that an important probiotic feature of *S. dentisani* is its anti-microbial properties, inhibiting the growth of important oral pathogens like *S. mutans, S. sobrinus*, or *Prevotella intermedia*. In addition, the killing of *Fusobacterium nucleatum* is unusual among oral probiotics and may provide an important beneficial effect, as this organism is responsible for a large number of co-aggregation patterns with many oral species and is considered the main “bridge” bacteria between early and late colonizers of dental plaque (Kolenbrander et al., 2002). Thus, its inhibition could contribute to impede the adhesion of pathogenic organisms that co-aggregate with this bacterium and by doing so hamper dental plaque development, whose maturity has been shown to considerably increase the drop in pH after a meal (Firestone et al., 1987). In addition, *F. nucleatum* itself has been associated with halitosis due to the production of volatile sulfur compounds (Krespi et al., 2006), and therefore its growth inhibition should be investigated in the future as a way to diminish the severity of this condition.

The different experiments performed in the current work suggest a peptidic nature of the inhibitory molecules, as the inhibitory effect was significantly reduced by proteinase treatment but was unaffected by peroxidase, indicating that the inhibition was not due to the production of hydrogen peroxide as it is common in other streptococci (Zhu and Kreth, 2012). This, together with the small size of the molecules responsible for the inhibition (<3 KDa) and the appearance of pores in the membrane of sensitive bacteria as identified by SEM, strongly suggest that the inhibition is caused by bacteriocins, and future work should be directed towards identifying and characterizing such antimicrobial peptides.

**FIGURE 5:**
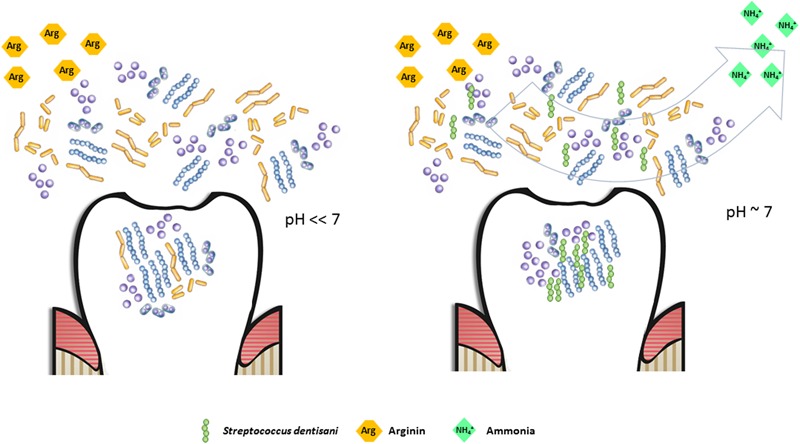
**Potential buffering effect in the oral cavity due to the enrichment of the dental plaque bacterial community with *Streptococcus dentisani*.** When arginine is available (either as an added prebiotic or as a product of peptide degradation) and *S. dentisani* is established on the tooth surfaces, the pH drop caused by the accumulation of organic acids can be buffered by the production of NH_4_^+^ through the arginolytic pathway, maintaining the pH close to neutrality (right panel). In the absence of *S. dentisani*, or if present at low proportions, the buffering effect would be diminished, with the consequent acidification and enamel damage. This would be the perfect scenario for the proliferation of acidogenic/cariogenic microorganisms (left panel).

Dental caries, as well as other oral diseases like periodontitis or halitosis are not considered typical infectious diseases in classical terms, as there is more than one species responsible for their etiology, the microbial consortia causing the disease varies considerably between individuals and even between lesions of the same patient, and pathogenic organisms normally can be isolated also from healthy individuals (Hajishengallis and Lamont, 2012; Simon-Soro et al., 2014; Camelo-Castillo et al., 2015; Marsh et al., 2015; Simón-Soro and Mira, 2015). For these reasons, antimicrobial properties of probiotics may not be sufficient for effectively preventing dental caries (ten Cate and Zaura, 2012) and the ability to restore the microbial ecological balance after pH acidification, for instance by alkali production, is a promising probiotic feature (Huang et al., 2015). Production of ammonia from urea or arginine by several oral bacteria has been shown to efficiently buffer salivary pH and has been associated to a reduction in caries risk (Reyes et al., 2014; Moncada et al., 2015). The analysis of the genome of *Streptococcus dentisani* revealed all genes from the arginolytic pathway, including the arginine deiminase and the carbamate kinase, a feature which appears to be common to all members of the cluster (Jensen et al., 2016). In addition, the agmatine deiminase gene, involved in the production of ammonia (Liu et al., 2012) was also present in both analyzed strains. In agreement with this, our data show that *S. dentisani* was able to efficiently buffer extracellular pH in the presence of arginine. Arginine is present in saliva in variable concentrations and it has also been added as a prebiotic to toothpaste, with strong clinical evidence for reducing enamel demineralization and buffering acidic pH (Yin et al., 2013; Santarpia et al., 2014). In addition, we have found in the genome of *S. dentisani* genes encoding for different aminopeptidases that can liberate arginine from peptides and proteins (Gonzales and Robert-Baudouy, 1996), which could then enter the arginolytic pathway. This is supported by the higher growth of the bacterium at pH 6 than at pH 6.5 in standard BHI growth medium (**Supplementary Figure S1**). Given that its optimal growth pH is neutral, and that the arginolytic pathway has been shown to be activated by low pH in other species (Liu et al., 2008), the higher growth rates at pH 6 are probably the result of protein degradation liberating arginine, which would allow the production of NH_4_, as well as the production of ATP derived from this metabolic route. These results also suggest that the full activation of the arginine degradation genes occurs at pH < 6.5, and the regulation of this pathway should be studied in the future. In **Figure 5**, we have illustrated the buffering effect that could take place in the oral cavity due to the enrichment of the dental plaque bacterial community with *S. dentisani*. When acidification occurs as a consequence of the consumption of dietary carbohydrates, and the pH drops to values around 6, *S. dentisani* would be able to activate the ADS system producing ammonia and, consequently, buffering the pH of its close environment.

The double beneficial action of *S. dentisani* (i.e., anti-microbial and anti-acid) makes it a promising probiotic bacterium. These benefits, together with its dental colonization capacity, derive to a big extent from its oral inhabitance. In addition, the choice of a dental plaque species as probiotic allows the quantification of the species in caries-free individuals, in order to use those levels as the appropriate administration dose, instead of using the dosages normally delivered for gut probiotics. In the current manuscript, we have quantified *S. dentisani* amounts in dental plaque for just two individuals and similar estimates for larger sample sizes could serve to accurately determine the dosage for an appropriate treatment.

For all these reasons, we propose the use of probiotics which are *active colonizers*, that is microorganisms which inhabit the site where the disease takes place, and that are isolated from healthy individuals. We believe that the administration of these organisms will maximize the chance of colonization and the potential beneficial effects for human and animal health. In the case of dental caries, we encourage the search of probiotic bacteria that are normal inhabitants of the human supragingival plaque in healthy individuals and propose the use of *S. dentisani* in clinical trials to test its potential in promoting oral health.

## Author Contributions

AM conceived the work, was implied in the analyses and interpretation of data and wrote the manuscript together with AL-L. AL-L, AC-C, and MF worked in the designing and performance of the experiments, data acquisition, analyses, and interpretation of the results. AS-S collected the plaque samples and made the bioinformatic analyses.

## Conflict of Interest Statement

The authors declare that the research was conducted in the absence of any commercial or financial relationships that could be construed as a potential conflict of interest.

## Footnotes

1 http://hmpdacc.org/

2 http://www.arb-silva.de/

3 www.ncbi.nlm.nih.gov/tools/primer-blast

## Supplementary Material

FIGURE S1 **Growth curves of *Streptococcus dentisani* strains 7746 (A)** and 7747 **(B)** in BHI medium at different starting pHs: 4.7 (red), 5.5 (orange), 6 (yellow), 6.5 (light blue), 7 (dark blue), and 7.5 (purple).
