# Supplementary figures and images for: Health-Associated Niche Inhabitants as Oral Probiotics: The Case of Streptococcus dentisani
Source: Front Microbiol. 2017 Mar 10;8:379. doi: 10.3389/fmicb.2017.00379 (PMC5344910; doi:10.3389/fmicb.2017.00379)

**A)**

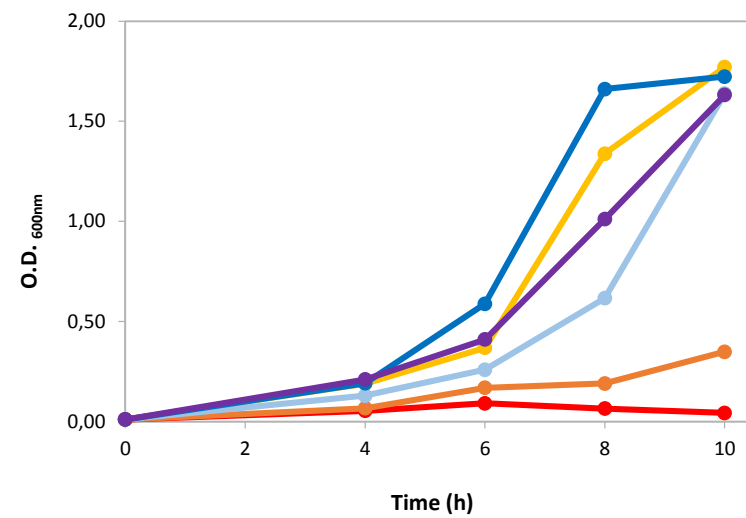

**B)**

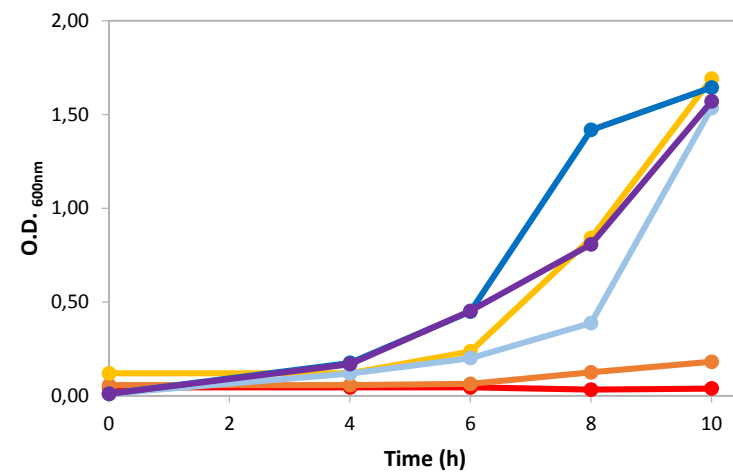

Supplement: FIGURE S1 — Growth curves of Streptococcus dentisani strains 7746 (A) and 7747 (B) in BHI medium at different starting pHs: 4.7 (red), 5.5 (orange), 6 (yellow), 6.5 (light blue), 7 (dark blue), and 7.5 (purple). [file Image_1.PDF]
